# In vitro radiosensitization of breast cancer with hypoxia‐activated prodrugs

**DOI:** 10.1111/jcmm.17486

**Published:** 2022-07-16

**Authors:** Radhika Aiyappa‐Maudsley, Lina Elsalem, Ali I. M. Ibrahim, Klaus Pors, Stewart G. Martin

**Affiliations:** ^1^ Nottingham Breast Cancer Research Centre, Biodiscovery Institute, School of Medicine University of Nottingham, University Park Nottingham UK; ^2^ Institute of Cancer Therapeutics, Faculty of Life Sciences University of Bradford Bradford UK; ^3^ Department of Molecular and Clinical Cancer Medicine University of Liverpool, William Henry Duncan Building Liverpool UK; ^4^ Jordan University of Science and Technology, Faculty of Medicine Department of Pharmacology Irbid Jordan; ^5^ Faculty of Pharmacy Al‐Zaytoonah University of Jordan Amman Jordan

**Keywords:** breast cancer, HAPs, hypoxia, hypoxia‐activated prodrugs, luminal, radiotherapy, TNBC, triple negative breast cancer

## Abstract

KP167 is a novel hypoxia‐activated prodrug (HAP), targeting cancer cells via DNA intercalating and alkylating properties. The single agent and radiosensitizing efficacy of KP167 and its parental comparator, AQ4N, were evaluated in 2D and 3D cultures of luminal and triple negative breast cancer (TNBC) cell lines and compared against DNA damage repair inhibitors. 2D normoxic treatment with the DNA repair inhibitors, Olaparib or KU‐55933 caused, as expected, substantial radiosensitization (sensitiser enhancement ratio, SER_0.01_ of 1.60–3.42). KP167 induced greater radiosensitization in TNBC (SER_0.01_ 2.53 in MDAMB‐231, 2.28 in MDAMB‐468, 4.55 in MDAMB‐436) and luminal spheroids (SER_0.01_ 1.46 in MCF‐7 and 1.76 in T47D cells) compared with AQ4N. Significant radiosensitization was also obtained using KP167 and AQ4N in 2D normoxia. Although hypoxia induced radioresistance, radiosensitization by KP167 was still greater under 2D hypoxia, yielding SER_0.01_ of 1.56–2.37 compared with AQ4N SER_0.01_ of 1.13–1.94. Such data show KP167 as a promising single agent and potent radiosensitiser of both normoxic and hypoxic breast cancer cells, with greater efficacy in TNBCs.

## 1 INTRODUCTION

Breast cancer is a leading cause of mortality among women worldwide, accounting for approx. 600,000 deaths in 2018.^1^ In the UK, breast cancer accounts for 15% of all new cancer cases with 11,399 deaths reported in 2018, making breast cancer the second leading cause of cancer‐related deaths.^2^ Clinically, patients diagnosed with ER/PgR positive (luminal) disease are associated with favourable prognosis and those with ER negative cancers (HER‐2 positive and triple negative breast cancers) are associated with increased risk of recurrence and death.^3^ ER negative cancers are also characterized by lower partial pressure of oxygen (pO_2_) and higher areas of hypoxia and anoxia, which contributes to therapy resistance and the tumour recurrence associated with such cancers.^4^ The presence of hypoxia, or low intracellular oxygen—pO_2_ < 10 mm Hg (<1%–2% O_2_), is present across all stages of breast cancer and is associated with reduced response to chemo‐ and radio‐therapy, tumour metastasis and poor survival.^5^ Molecular oxygen is an effective radiosensitiser, contributing to the cytotoxic action of radiation by reacting with low‐linear energy transfer (LET) (X‐ray) radical induced DNA damage, leading to the formation of peroxinitrite DNA lesions which are unrepairable, resulting in cell damage and death, known as the oxygen fixation hypothesis (OFH).^6^, ^7^ The presence of molecular oxygen is crucial to enhance the therapeutic, or biological, efficacy of low‐LET x‐radiation and is referred to as the oxygen enhancement ratio (OER).^8^ Research spanning over several decades has focused on increasing radiosensitivity of the relatively radioresistant hypoxic cancer cells found in tumours, using hypoxia‐activated prodrugs (HAPs) that are chemically active under low‐oxygenated conditions and demonstrate cytotoxicity to hypoxic cells.^9^, ^10^

**FIGURE 1:**
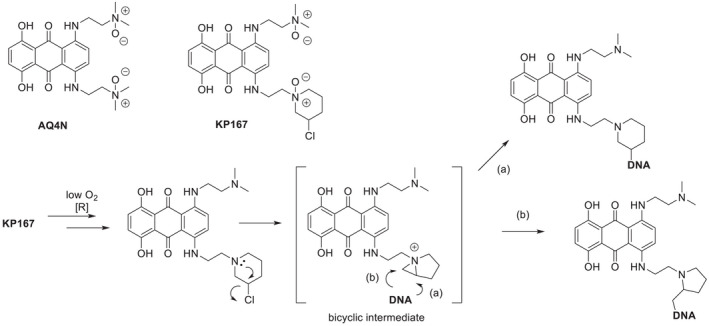
Chemical structures and proposed hypoxic bioactivation of KP167. Enzymatic reduction [R] of KP167 under low oxygen or hypoxic conditions may result in the formation of two metabolites (a) and (b) capable of causing DNA adducts

AQ4N is a di‐*N*‐oxide aliphatic amino anthraquinone, that is efficiently bio‐reduced in a hypoxic environment by the cytochrome P450 (CYP) family of reductases into the DNA binding, topoisomerase II (TOPO II) inhibitor, AQ4. The ‘orphan’ CYP enzymes, CYP2S1 and CYP2W1 have been reported to catalyse the hypoxic reduction of AQ4N more efficiently compared with the previously identified CYP enzymes and inducible nitric oxide synthase (iNOS).^12^ The reduced metabolite, AQ4, interacts with DNA non‐covalently, and inhibits TOPO II in hypoxic cells, as they re‐enter the cell cycle during reoxygenation, with a given dose of chemo‐ or radio‐therapy killing the outer oxygenated counterparts.^11^ Some TOPO II inhibitors, such as doxorubicin, are currently used in breast cancer treatment and function as potent anti‐cancer agents as they prevent unwinding of DNA strands during replication and repair, resulting in cell death through the generation of cytotoxic DNA double‐strand breaks.^13^, ^14^ AQ4N is an effective radio‐^15^, ^16^ and chemo‐sensitizer, sensitizing the effects of cisplatin and cyclophosphamide to induce greater tumour cell death. When combined with mitoxantrone, AQ4N has the potential to improve mitoxantrone penetration and localisation to the more hypoxic areas of the tumour.^17^, ^18^, ^19^ This enhancement in the anti‐cancer efficacy of either chemo‐ or radio‐therapy, when combined with AQ4N is due to the targeting of both hypoxic and oxic populations of the tumour.^20^ The development of AQ4N stalled at early clinical evaluation despite clinical proof‐of‐concept being demonstrated. More recently, additional analogues, such as OCT1002, are showing promising chemosensitizing properties when combined with bicalutamide in prostate cancer.^21^ The newly reported novel analogue, KP167, is an anthraquinone‐based di‐*N*‐oxide prodrug which, unlike AQ4N, possesses capacity for covalently adducting DNA (Morral J., Aiyappa R., Sadiq M., Abdallah Q. M., Grau L., Puyol M. D., Shnyder S. D., Phillips R. M., Patterson L. H., Martin S. and Pors K., unpublished data). This agent is preferentially activated under hypoxic conditions, generating a cytotoxic metabolite via a presumed bicyclic electrophile.^22^ The proposed hypoxic bioactivation of KP167 is shown in Figure 1, along with chemical structures of AQ4N and KP167.

Initial work in colon (HT29) and breast (MDAMB‐231) cancer cell lines has shown that KP167 is antiproliferative under hypoxic conditions (0.1% O_2_), and improves radiosensitivity of spheroid cultures (Morral J., Aiyappa R., Sadiq M., Abdallah Q. M., Grau L., Puyol M. D., Shnyder S. D., Phillips R. M., Patterson L. H., Martin S. and Pors K., unpublished data). However, it remains to be seen if the agent is activated in other conditions where endogenous reductase activity may be important. For example, the CYP enzymes are associated with ER positive cancers mainly through their involvement in the oxidation and metabolism of oestrogen.^23^ It is yet to be determined if tumours that express CYPs (i.e. luminal cancers) may be sensitive to HAPs that are reliant on CYP2S1 and CYP2W1 for metabolism, regardless of hypoxic conditions. Furthermore, the radiosensitizing efficacy of KP167 has not been established in the different breast cancer subtypes. The current study sought to examine the single agent and radiosensitizing effect of KP167 in 3D spheroid, 2D normoxia and 2D hypoxia cultured breast cancer cell line models of differing phenotypes, that is luminal versus TNBC. Results obtained were compared with the parental drug, AQ4N and well‐established radiosensitisers: the PARP inhibitor, Olaparib and ATM inhibitor, KU‐55933.

## 2 MATERIALS AND METHODS

### 2.1 Cell culture

The luminal (MCF‐7 and T47D) and TNBC cell lines, (MDAMB‐231, MDAMB‐468 and MDAMB‐436 [BRCA‐1 deficient]) and SW480 colon carcinoma cells were initially procured from the American Type Culture Collection (ATCC) and cultured in specific media formulations listed in Table S1. All cell lines were used within a passage window of 15 and maintained in a humidified incubator with 5% CO_2_ at 37°C. Cells were authenticated using the Promega Powerplex® 16‐short‐tandem‐repeat system and regularly screened for Mycoplasma using the MycoProbe® Mycoplasma Detection Kit (R&D Systems). To culture spheroids, 0.5‐2 × 10^4^ cells in 100 μl medium (supplemented with 5 μl matrigel [BD] for MDAMB‐231 and MDAMB‐468) were seeded in 96‐well ultra‐low attachment round‐bottomed plates (Corning) and aggregated by centrifugation at 2000 r.p.m for 10 min. Plates were incubated at 37°C, 5% CO_2_ for 48 h to allow spheroid formation. 100 μl of fresh culture media was added the next day, and spent media was replaced every 48 h. For 2D hypoxia experiments, 4 × 10^5^ cells were seeded in T‐25 cm^2^ flasks and allowed to adhere in a normoxic incubator (21% O_2_) for 3 h. Following attachment, cells for hypoxia experiments were placed in a HypoxyLab™ (Oxford Optronix) hypoxia workstation which was maintained at 1% O_2_ levels for 24 h. SW480‐2 W1 cells stably expressing CYP2W1 were generated using the Flp‐In system (Invitrogen) details of which are given in Travica et al.^24^

### 2.2 Drug preparation

AQ4N (Sigma) and KP167 were reconstituted in distilled water to a stock concentration of 10 mM. Olaparib (Santa Cruz Biotechnology) and KU‐55933 (Tocris Biosciences) were reconstituted in DMSO to a stock concentration of 10 mM. All stock solutions were aliquoted and stored at −20°C for a maximum of 3 months and repeat freeze thaw cycles avoided. A new batch of drugs was always tested for similar response between new and old to ensure consistency and to minimize batch–batch variability.

### 2.3 Drug penetration

To assess drug penetration in 3D cultures representative MCF‐7 and MDAMB‐231 spheroids (>500 μm) were treated with 5 μM AQ4N or KP167 (fluorescent due to the presence of the anthraquinone chromophore)^25^, ^26^ for 8 h. Following treatment, spheroids were fixed in 4% PFA followed by treatment with 100 mM glycine. Spheroids were resuspended in ice‐cold methanol and pipetted onto slides, allowed to air dry and mounted in ProLongTM antifade mounting medium (Invitrogen). Images (10× magnification) were obtained by performing Z stack by laser scanning confocal microscopy (Leica TCS SPE). AQ4N and KP167 were visualized between 650 and 800 nm (λ excitation‐647 nm).^27^ Negative controls with DMSO did not give any detectable signals. Data represent two independent experiments, with each containing 30 spheroids.

### 2.4 MTT assay

SW480‐2 W1 cells, used to assess influence of CYP2W1 on drug response, were grown both in normoxia and in hypoxia (0.1% O_2_), in triplicate and treated with various concentrations of KP167 (0.01–100) μM for 24 h. The anti‐proliferative efficacy of KP167 treatment was determined using the MTT assay, according to the manufacturer's guidelines (Biomedica).

### 2.5 Western Blot

Cells grown in 2D normoxia or in 2D hypoxia were lysed in RIPA Buffer (Sigma), supplemented with 1× phosphatase and protease inhibitor cocktail, and EDTA (ThermoFisher Scientific) and centrifuged at 13000 *g* for 15 min at 4°C to obtain protein extracts. Extracts were subjected to SDS‐Page and, following separation, proteins electroblotted onto a nitrocellulose membrane. After blocking, membranes were probed with anti‐P450 (ab180597), anti‐CYP2S1 (ab69650), anti‐CYP2W1 (ab76666), anti‐CA9 (ab00414‐1.1) or β‐actin (ab8226) (loading control). Membranes were probed with appropriate secondary antibodies (Li‐COR or horse radish peroxidase [HRP]‐conjugated) (DAKO). Blots were scanned using an Odyssey® FC Imager (LI‐COR Biosciences) and quantified using Image Studio Version 4.1 (LI‐COR Biosciences). Data presented graphically represent the mean of normalized protein expression to β‐actin ± SD of three independent experiments, with separate cell lysates from different passage numbers of cells.

### 2.6 Single agent clonogenic survival assays

Preplated cells at low seeding density were treated with 10 μM Olaparib or KU‐55933 for 24 h then incubated for colony formation. HAPs‐2D normoxia: 1 × 10^5^ cells/well maintained in normoxia were treated with various concentrations of AQ4N or KP167 (5–45) μM for 24 h. 2D hypoxia: 1 × 10^5^ cells/well were maintained in a HypoxyLab™ workstation at 1% O_2_ for 24 h, then treated with various concentrations of AQ4N (0.01–10) μM or KP167 (0.15–10) μM for a further 24 h under hypoxia. Spheroids (>500 μm) were treated with various concentrations of AQ4N (0.1–50) μM or KP167 (0.01–45) μM for 96 h. Drug‐media was refreshed at the 48‐hour time‐point. Following HAP treatment, 2D and 3D grown cells were trypsinised into single cell suspensions and plated at low density into T‐25 cm^2^ tissue culture flasks for 14 (MCF‐7, MDAMB‐231 and MDAMB‐468) or 21 days (T47D and MDAMB‐436) to allow colony formation. Colonies were fixed and stained with 0.5% crystal violet. Colonies containing more than 50 cells were scored as survivors. Dose response curves were constructed by plotting surviving fraction versus drug concentration (μM). IC_50_ doses were calculated using GraphPad Prism 7 software. Data represent the average SF ± SD of three independent experiments, with each experiment containing six parallel sets.

### 2.7 Cell irradiation

Cells were irradiated using a RS225 Xstrahl X‐ray cabinet irradiation system (Xstrahl Limited, UK) with single doses of 2, 4, 6, or 8 Gy. X‐rays were delivered at 195 kV, 10 mA, with a dose rate of 0.87 Gy/min. The cabinet was fitted with a 0.5 mm copper filter and used a 48.4 cm focus‐to‐skin distance. Sham irradiated cells were used as controls. For DNA repair inhibitor‐radiation combination experiments, pre‐plated cells at low density were treated with 10 μM KU‐55933/Olaparib for 1‐hour pre‐ and 16–20 h post‐irradiation. After media change, flasks were transferred to a dedicated clonogenic incubator for colony formation. HAP‐radiation combination experiments: 2D and 3D cells were treated with the IC_50_ of AQ4N or KP167 (2D normoxia and spheroids for 96 h and 2D hypoxia cells for 24 h). Following irradiation, cells at low density were plated for conventional 2D clonogenic assay as described above. The SF of drug‐radiation combination experiments was corrected for independent cytotoxic effects of HAPs/DNA repair inhibitors. Dose–response curves were plotted as a function of radiation dose on a log/linear plot using the linear quadratic (LQ) model and parameters calculated using the open source survival calculation programme, CS‐CAL developed by the German Cancer Research Centre and available at – http://angiogenesis.dkfz.de/oncoexpress/software/cs‐cal/index.htm. The SER at 1% survival (SER_0.01_) was calculated as the ratio of radiation doses in the absence to the presence of the drug which produces the same biological effect.^28^ SER values greater than 1.00 indicate radiosensitization. Data represent the mean SF ± SD of three (or two for 2D hypoxia) independent experiments, with each experiment containing six parallel sets.

## 3 RESULTS

### 3.1 Effect of control DNA repair inhibitors on radiosensitivity in 2D normoxia

**FIGURE 2:**
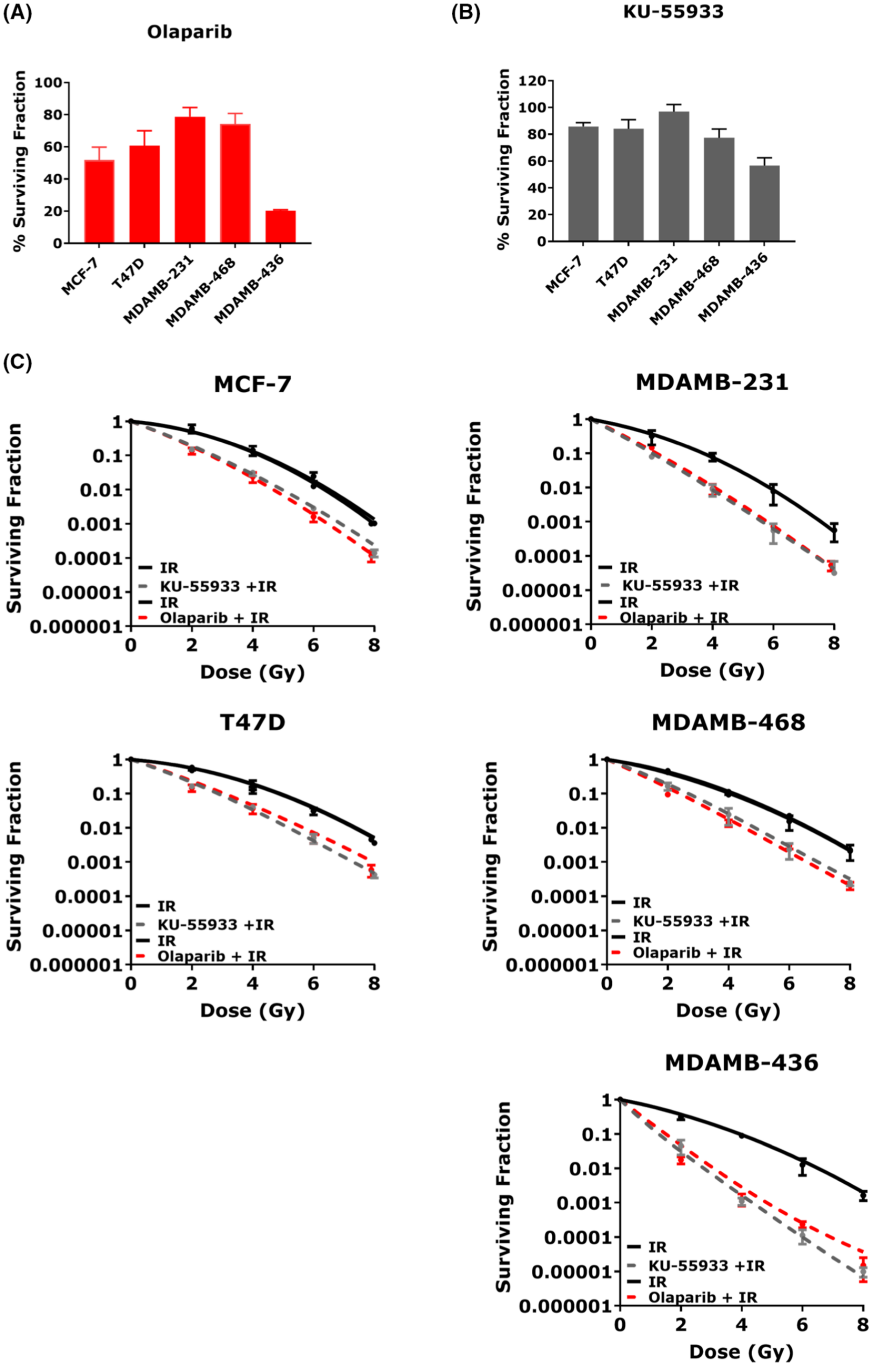
KU‐55933 and Olaparib treated breast cancer cells. (A) Single agent Olaparib (B) Single agent KU‐55933 (C) Radiation survival curves of Olaparib and KU‐55933. PEs of individual cell lines were: MCF‐7 – 45 ± 4.72%; T47D – 15 ± 2.64%; MDAMB‐231 – 49 ± 4.72%; MDAMB‐468 – 44 ± 2.08%; and MDAMB‐436 – 19 ± 2.51%. Data represent the mean SF ± SD of three independent experiments, with each experiment containing six parallel data sets

Olaparib and KU‐55933, inhibitors of PARP and ATM, respectively, are well established radiosensitisers^29^, ^30^ and were used as positive controls and comparators to assess efficacy of HAPs. Single agent Olaparib (Figure 2A) and KU‐55933 (Figure 2B) treatments were associated with low levels of cytotoxicity in the repair proficient MCF‐7, T47D, MDAMB‐231 and MDAMB‐468 cells. As expected,^30^, ^31^ Olaparib induced a synthetic lethal response in MDAMB‐436 cells due to their BRCA‐1/2 deficiency and resulted in an 80% decrease in colony formation compared with 44% with KU‐55933. Both agents substantially increased radiosensitivity across all breast cancer phenotypes with SER_0.01_ ranging 1.61–2.35 and 1.60–3.42, respectively (Figure 2C). Olaparib radiosensitized both BRCA‐1 proficient and deficient cells, with a surviving fraction at 2Gy (SF2) radiation alone versus drug + radiation of 49% versus 18%, respectively (*p* < 0.00001) in MCF‐7, 49% versus 22% (*p* = 0.011) in T47D, 31% versus 10% (*p* = 0.002) in MDAMB‐231, 38% versus 13% (*p* = 0.0006) in MDAMB‐468 and 28% versus 2% (*p* = 0.0001) in MDAMB‐436 cells. SF2 in radiation alone control cells were higher than KU‐55933 treated cells, indicating substantial radiosensitization, that is 41% versus 19% (*p* = 0.02) in MCF‐7, 55% versus 21% (*p* = 0.007) in T47D, 36% versus 10% (*p* = 0.020) in MDAMB‐231, 43% versus 13% (*p* < 0.001) in MDAMB‐468. As with single agent treatment, the greatest radiosensitization was seen in the BRCA‐1 deficient MDAMB‐436 cells, 35% versus 3% (*p* = 0.003). Other radiobiologic parameters including *α*, *β* and *α*/*β* ratios are given in Table S2.

### 3.2 Response to CYP enzymes

**FIGURE 3:**
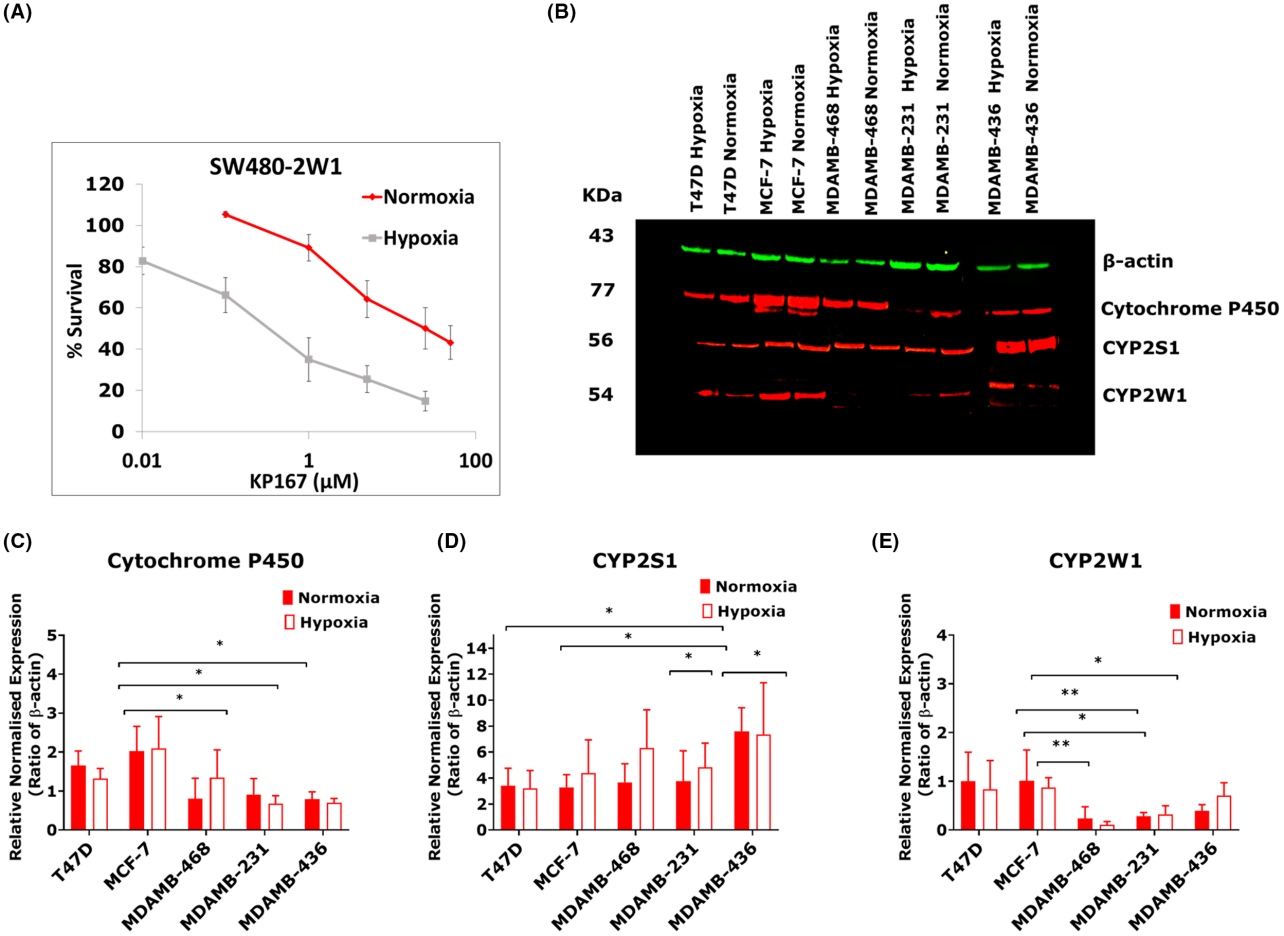
Bioactivation by CYPs. (A) Bioactivation of KP167 by CYP2W1 in normoxic and hypoxic conditions (0.1% O_2_). Data represent mean of three independent experiments. (B) Representative P450, CYP2S2, CYP2W1 and β‐actin Western blot images. Quantified expression of (C) Cytochrome P450 (D) CYP2S1 and (E) CYP2W1. Red bands indicate cytochrome P450, CYP2S1, CYP2W1 expression and green bands represent β‐actin. Data in the bar charts represent the mean of normalized protein expression to β‐actin ± SD of three independent experiments, with separate cell lysates from different passage numbers of cells, which are analysed using Student's *t*‐test. *p < 0.05, **p < 0.01

The orphan CYP, CYP2W1 has been reported to be involved in the reduction of AQ4N to AQ4, at a higher rate of approximately 12 mol of substrate per mole of enzyme per minute.^12^ To assess if CYP2W1 is involved in the bio‐reductive activation of KP167 MTT assay was carried out in the SW480‐CYP2W1 transfected cell line.^24^ Data show (Figure 3A) that KP167 was more cytotoxic and decreased proliferation in SW480‐W1 cells maintained in hypoxia (0.1% O_2_) with an IC_50_ of 0.32 ± 0.08 μM compared with an IC_50_ of 25 ± 5.5 μM in normoxic conditions. Such data suggest that that CYP2W1 enzymes bioreduce KP167 preferentially in hypoxic conditions compared with normoxia. To assess the endogenous expression of CYP enzymes in the luminal and TNBC cells, protein expression was assessed by Western Blot (Figure 3B). Cytochrome P450 (Figure 3C) and CYP2W1 (Figure 3E) expression was highest in the luminal MCF‐7 and T47D cells, compared with the triple negative MDAMB‐231, MDAMB‐468, MDAMB‐436 cells. Overall, CYP2S1 (Figure 3D) expression was higher in the TNBC cell lines, especially in the MDAMB‐436 cells.

### 3.3 Single agent HAP cytotoxicity

**FIGURE 4:**
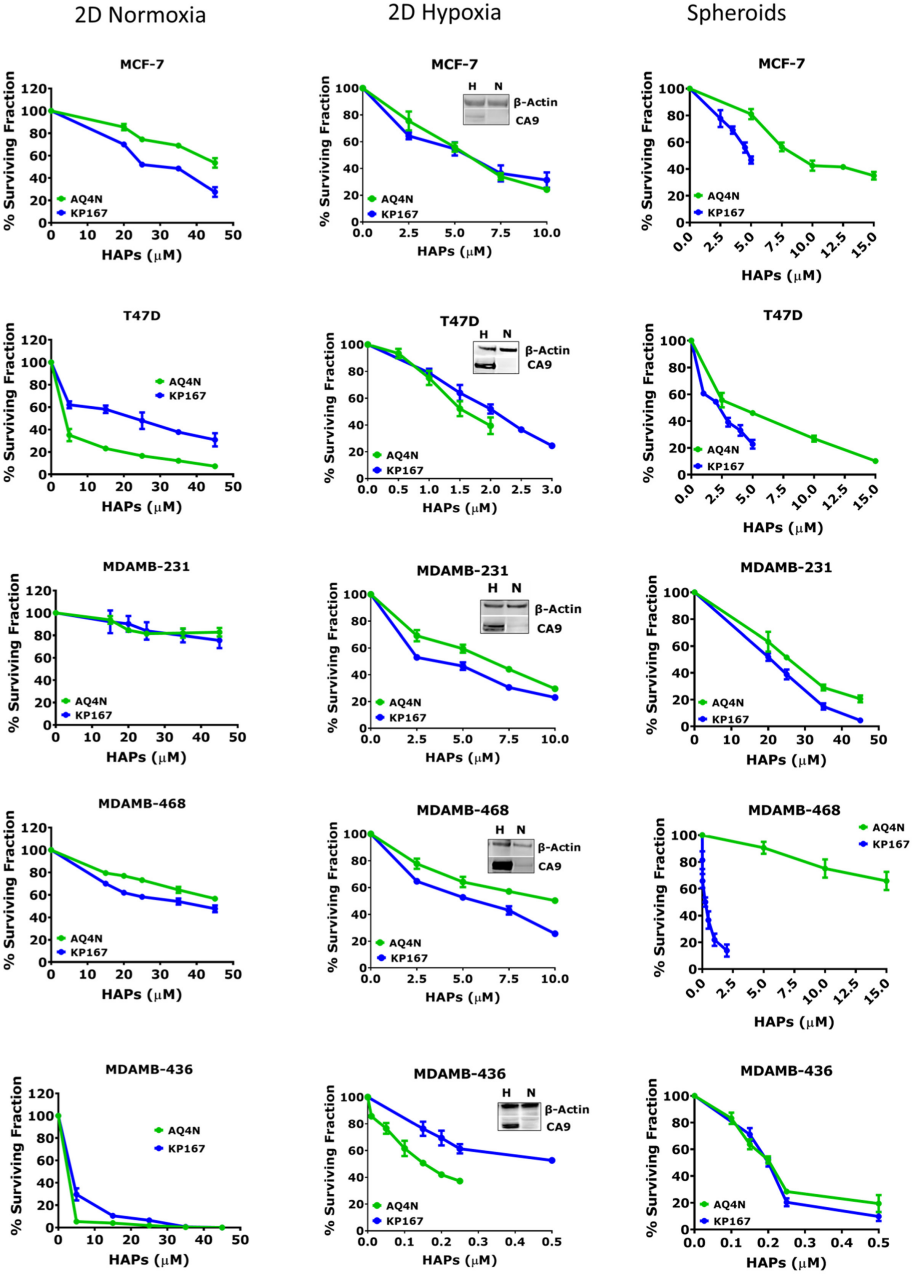
Effect of HAPs on clonogenic survival in 2D normoxia, hypoxia and spheroid cultures. PEs of individual cell lines were: MCF‐7 – 55 ± 3.54%; T47D – 21 ± 2.50%; MDAMB‐231 – 64 ± 4.34%; MDAMB‐468 – 50 ± 3.13%; MDAMB‐436 – 21 ± 5.41%. Inset image in 2D hypoxia graphs shows the representative blot for the expression of CA9 in normoxia (N) and hypoxia (H) with β‐Actin used as a loading control. Data represent the mean ± SD of three independent experiments, with each experiment containing six parallel data sets

The single agent cytotoxic effect of AQ4N and KP167 was assessed in breast cancer cells grown in 2D normoxia, 2D hypoxia and 3D spheroid cultures to assess the potency of these agents both in the presence and absence of oxygen (Figure 4). In 2D normoxia, cells demonstrated a differential response to treatment with most cell lines not achieving IC_50_ levels within the concentrations (5–45 μM) tested. HAP treatment was highly toxic in MDAMB‐436 cells which demonstrated total cell kill, even at the lowest concentration of 5 μM, compared with the highly resistant MDAMB‐231 cells with an IC_50_ > 45 μM. In 2D hypoxia, the expression of CA9 was present in all breast cancer cells exposed to hypoxia and absent in cells grown in normoxic culture conditions, confirming a hypoxic response. KP167 was more cytotoxic in 2D hypoxic cultures with IC_50's_ of 0.37–4.9 μM compared with AQ4N IC_50's_ of 0.15–9.5 μM. Spheroids of more than >500 μm in diameter were used to assess efficacy of HAPs. Spheroid morphology and growth pattern over an 8‐day culture is shown in Figure S1. In 3D cultures, KP167 IC_50's_ ranged between 0.14 and 12.8 μM, which was lower than the AQ4N IC_50's_ of 0.2–22.8 μM. In 3D culture, KP167 IC_50_ values were 1‐2‐fold lower than the parental drug AQ4N in most cell lines, and 100‐fold lower in the MDAMB‐468 cells. Fluorescent Z‐stack imaging of spheroid cross‐sections, shown in Figure S2, show that both HAPs demonstrated similar levels of penetration across different layers of the spheroid cultures, suggesting that any effect seen was not due to sub‐optimal drug penetration. The increase in the single‐agent cytotoxic effect of KP167 may be due to its ability to bind DNA covalently and kill both proliferating and quiescent tumour cells by adducting covalently with DNA, as opposed to AQ4N which functions only as a TOPO II inhibitor in proliferating cells. AQ4N and KP167 treatments were more cytotoxic in spheroids and 2D hypoxic cultures, with lower IC_50's_ in most cell lines compared with cells grown in 2D normoxia. Both agents were cytotoxic in T47D and MDAMB‐436 cells across all three conditions, regardless of oxygen availability. Although further studies are required to identify reasons for these observations, the inherent double‐strand break repair deficiency along with increased CYP2S1 in the BRCA‐1 deficient MDAMB‐436 cells and the increased expression of CYP2W1 in T47D cells (assessed by Western blot), may explain, in part, the sensitivity of these cells to HAPs based on DNA‐affinic anthraquinone pharmacophores such as AQ4N and KP167. Tabulated IC_50_ doses are supplied in Table 1.

**TABLE 1:** IC_50_ of AQ4N and KP167 in 2D normoxia, 2D hypoxia (1% O_2_) and 3D spheroid cultures

Cell line	AQ4N (μM)			KP167 (μM)		
	2D Normoxia	2D Hypoxia	3D Spheroids	2D Normoxia	2D Hypoxia	3D Spheroids
MCF‐7	43.6 ± 18.7	5.2 ± 1.9	8.5 ± 2.9	26.2 ± 10.0	4.9 ± 0.95	6.1 ± 0.4
T47D	<5	2.0 ± 0.1	4.4 ± 0.5	19.4 ± 6.9	2.2 ± 0.49	1.9 ± 0.6
MDAMB‐231	>45	6.1 ± 2.7	21.1 ± 4.9	>45	3.4 ± 1.24	12.8 ± 1.3
MDAMB‐468	>45	9.5 ± 0.8	22.8 ± 1.1	32.6 ± 4.2	4.8 ± 0.31	0.2 ± 0.01
MDAMB‐436	<5	0.15 ± 0.03	0.2 ± 0.01	<5	0.37 ± 0.01	0.14 ± 0.01

*Note*: The IC_50_ values are means of at least three independent experiments and were calculated using GraphPad prism software.

Abbreviations: μM, micromolar.

### 3.4 Effect of HAPs on radiosensitivity in 2D normoxia

**FIGURE 5:**
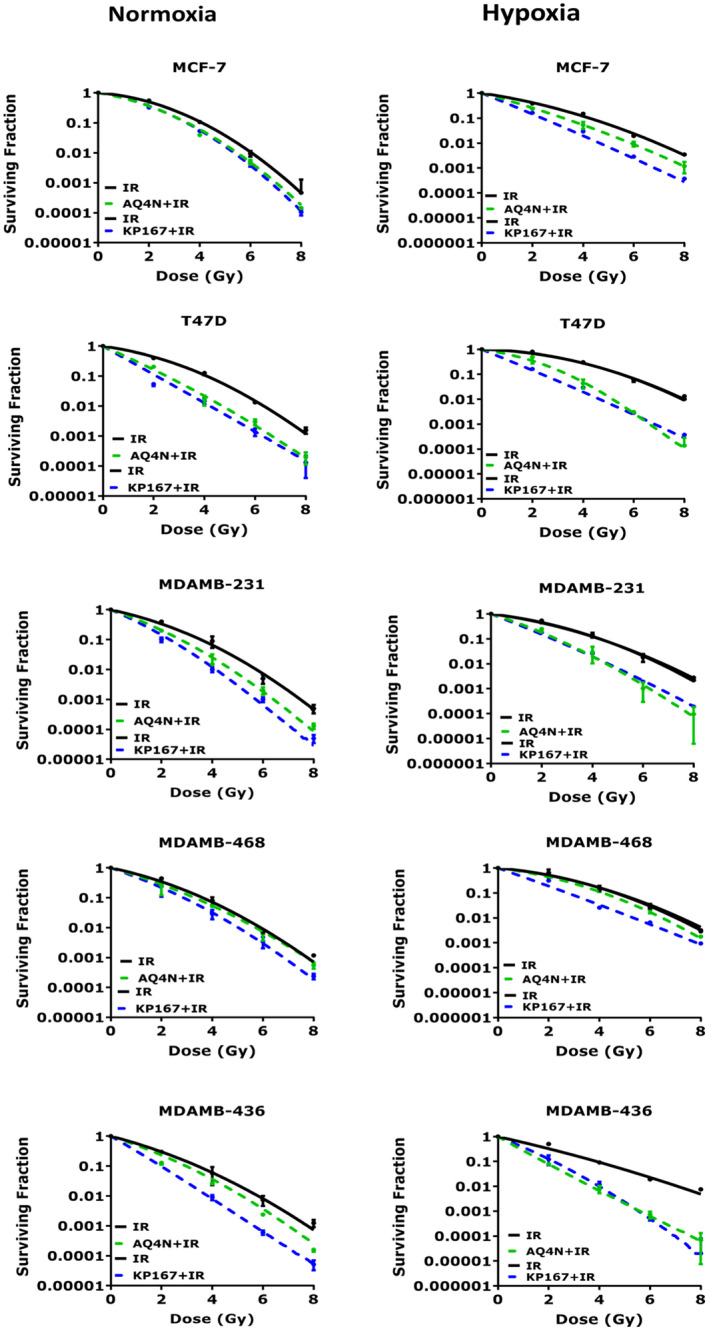
HAP induced radiosensitivity in 2D normoxia and hypoxia. PEs of individual cell lines in normoxia and hypoxia were as follows: MCF‐7 – 40 ± 6.97% and 42 ± 0.5%; T47D – 19 ± 1.34% and 12 ± 1.0%; MDAMB‐231 – 56 ± 4.87% and 33 ± 1.5%; MDAMB‐468 – 46 ± 5.48% and 42 ± 1.5%; MDAMB‐436 – 32 ± 2.55% and 16 ± 3.6%. Data represent the mean SF ± SD of two independent experiments, with each experiment containing six parallel data sets

The radioresponse of cells treated with AQ4N or KP167 was assessed in 2D normoxia to investigate if doses that were cytotoxic in spheroid cultures, were able to induce radiosensitivity in normoxic cells. Radiosensitization in 2D normoxia was cell line dependent (Figure 5). Control, radiation alone, SF2 values versus AQ4N treated + radiation SF2's was 36% versus 22% (*p* = 0.002) in MCF‐7, 26% versus 10% (*p* = 0.027) in T47D, 25% versus 12% (*p* = 0.007) in MDAMB‐231, 32% versus 27% (*p* = 0.0008) in MDAMB‐468 and 62% versus 12% (*p* = 0.006) in MDAMB‐436 cells with SER_0.01_'_s_ of 1.11–1.29. The greatest increase in radiosensitivity was seen in T47D cells with an SER_0.01_ of 1.64 ± 0.27. KP167 was a more effective radiosensitiser than AQ4N with SER_0.01_'_s_ of 1.21 ± 0.05 in MCF‐7, 1.93 ± 0.09 in T47D, 1.43 ± 0.46 in MDAMB‐231, 1.32 ± 0.19 in MDAMB‐468 and 1.68 ± 0.08 in MDAMB‐436 cells. Mean SF2 values in control radiation alone treated cells were higher than drug‐radiation combination groups, that is 50% versus 36% in MCF‐7, 44% versus 2% in T47D, 33% versus 14% in MDAMB‐231, 34% versus 21% in MDAMB‐468 and 23% versus 8% in MDAMB‐436 cells than KP167 treated cells. Greater radiosensitization was seen in the luminal T47D and BRCA‐1 deficient MDAMB‐436 cells. Radiobiologic parameters of normoxia are summarized in Table S3.

### 3.5 Effect of HAPs on radiosensitivity in 2D hypoxia

Prior to 2D hypoxia drug‐radiation combination experiments, the radioresponse of breast cancer cells grown in 2D hypoxia was assessed, to identify if hypoxia influences radiosensitivity. As shown in Figure S3 and Table S4, hypoxia substantially increased radioresistance with oxygen enhancement ratio (OER)_0.01_ of 1.69 ± 0.22 in MCF‐7, 1.44 ± 0.20 in T47D, 1.55 ± 0.01 in MDAMB‐231, 1.53 ± 0.07 in MDAMB‐468 and 1.30 ± 0.03 in MDAMB‐436 cells. Therefore, HAP treatment must effectively counter the induced radioresistance of hypoxic cells to achieve greater cell kill. This is contrast to normoxically active agents that can only function to sensitize, but not counter the radioresistance of hypoxic cells. The radiosensitizing efficacy of both AQ4N and KP167 was assessed by treating 2D hypoxia cultured breast cancer cells with the clonogenic IC_50_ values shown in Table 1. Figure 5 shows that the radiosensitization by AQ4N ranged from marginal to substantial, with effects more pronounced in the luminal T47D cells. Compared with control, radiation alone, treated cells, the mean SF2 values were significantly reduced in combined AQ4N/radiation treated cells, that is 41% versus 25% (*p* = 0.020) in MCF‐7, 68% versus 35% (*p* < 0.0001) in T47D, 45% versus 18% (*p* = 0.008) in MDAMB‐231 and 26% versus 7% (*p* = 0.0003) in MDAMB‐436 cells, but not in MDAMB‐468 cells with SF2 of 52% versus 43% (*p* = 0.225). SER_0.01_ values were 1.30 ± 0.08 in MCF‐7, 1.74 ± 0.32 in T47D, 1.69 ± 0.11 in MDAMB‐231, 1.13 ± 0.10 in MDAMB‐468 and 1.94 ± 0.25 in MDAMB‐436 cells. The radiosensitivity obtained was, however, substantially higher with KP167 across both luminal and TNBC phenotypes, with SER_0.01_ values of 1.56 ± 0.12 in MCF‐7, 2.37 ± 0.11 in T47D, 1.75 ± 0.04 in MDAMB‐231, 1.72 ± 0.02 in MDAMB‐468 and 1.81 ± 0.33 in MDAMB‐436 cells. Compared with controls, mean SF2 values in KP167 treated cells were significantly reduced, that is 43% versus 19% (*p* = 0.032) in MCF‐7, 69% versus 14% (*p* = 0.001) in T47D, 43% versus 15% (*p* = 0.003) in MDAMB‐231, 50% versus 18% (*p* = 0.0002) in MDAMB‐468 and 32% versus 12% (*p* = 0.003) in MDAMB‐436 cells. Overall, even though hypoxia induced radioresistance, the radiosensitivity observed in 2D hypoxia was higher than in 2D normoxia, suggesting that KP167, like AQ4N, may be more effective and reliant on hypoxia for activation. Radiobiologic parameters are summarized in Table S5.

### 3.6 Effect of HAPs on radiosensitivity in 3D spheroids

**FIGURE 6:**
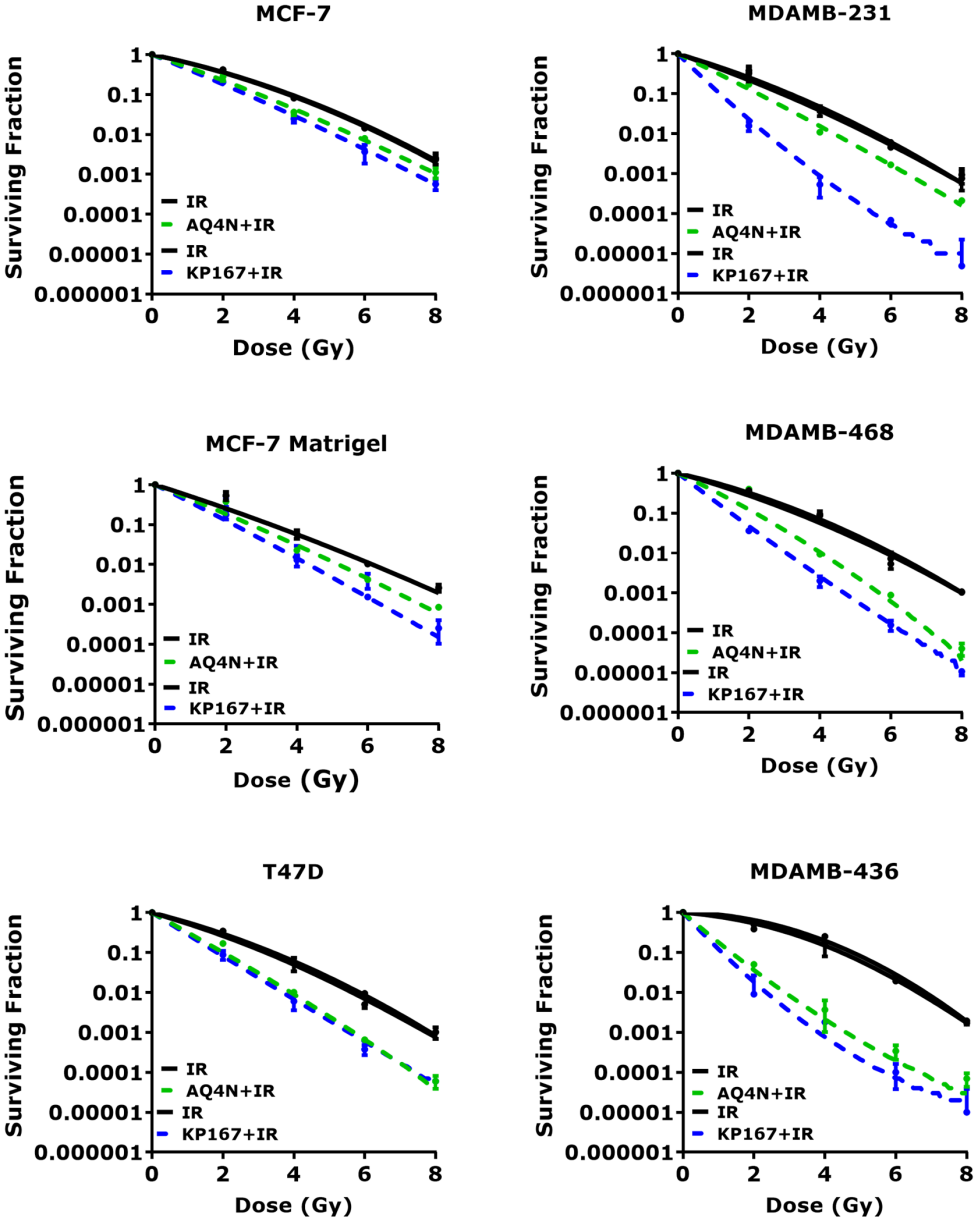
HAP induced radiosensitivity in 3D spheroids. PEs of individual cell lines were as follows: MCF‐7 – 33 ± 5.51%; T47D – 22 ± 2.64%; MDAMB‐231 – 74 ± 1.93%; MDAMB‐468 – 35 ± 1.52%; and MDAMB‐436 – 6 ± 0.57%. Data represent the mean SF ± SD of three independent experiments, with each experiment containing six parallel data sets

Spheroid culture is a well‐established 3D model bridging the gap between conventional 2D cell culture and in vivo studies, as the models are more reflective of in vivo tumour growth and drug responses.^31^ The radioresponse of breast cancer spheroids was assessed by treatment with the clonogenic IC_50_ of AQ4N or KP167 for 96 h (Figure 6). The greatest radiosensitivity was seen using the 3D spheroid models. AQ4N treatment increased radiosensitivity across all breast cancer phenotypes, with effects more pronounced in the MDAMB‐436 cells, with SER_0.01_ of 3.33 ± 0.18 compared with 1.30 ± 0.08 (MCF‐7), 1.58 ± 0.06 (T47D), 1.44 ± 0.04 (MDAMB‐231) and 1.64 ± 0.08 (MDAMB‐468). Mean SF2 values in control, radiation alone, cells were significantly higher compared with combined AQ4N/radiation treated cells, that is 36% versus 22% (*p* = 0.002) in MCF‐7, 26% versus 10% (*p* = 0.027) in T47D, 25% versus 13% (*p* = 0.007) in MDAMB‐231, 32% versus 27% (*p* = 0.0008) in MDAMB‐468 and 62% versus 12% (*p* = 0.006) in MDAMB‐436 cells.

Compared with 2D normoxic and hypoxic culture conditions, substantially increased radiosensitization was seen with KP167 across all breast cancer spheroid models, with the greatest increase seen in TNBC cells with SER_0.01_ of 2.53 ± 0.06 in MDAMB‐231, 2.28 ± 0.24 in MDAMB‐468 and 4.55 ± 0.24 in MDAMB‐436 compared with 1.46 ± 0.66 in MCF‐7 and 1.76 ± 0.20 in T47D cells. The mean SF2 values in KP167 treated spheroids were significantly lower than controls, that is 35% versus 18% (*p* = 0.002) in MCF‐7, 23% versus 6% (*p* = 0.009) in T47D, 21% versus 2% (*p* = 0.001) in MDAMB‐231, 27% versus 4% (*p* = 0.0002) in MDAMB‐468 and 64% versus 1% (*p* = 0.0001) in MDAMB‐436 cells. Other radiobiologic parameters including *α*, *β* and *α*/*β* ratios are given in Table S6. Overall SER value comparison values for DNA repair inhibitors and HAPs is supplied in Table S7. In all culture conditions, KP167 was able to induce greater radiosensitization in most cell lines compared with AQ4N. The greatest increase in radiosensitization was seen in the triple negative breast cancer 3D models of MDAMB‐231, MDAMB‐468 and MDAMB‐436 cells. This increase was greater compared with well‐established radiosensitisers such as KU‐55933 and Olaparib.

Matrigel has been reported, in certain studies, to alter sensitivity to drugs, with addition of an extracellular matrix (ECM) either enhancing drug efficacy or promoting drug resistance.^32^, ^33^, ^34^ To assess whether the addition of matrigel altered response in MDAMB‐231 and MDMAB‐468 spheroids to KP167, an additional experiment was conducted in MCF‐7 cells. MCF‐7 cells were formed into spheroids with and without matrigel (MCF‐7 + M and MCF‐7, respectively), and response to AQ4N or KP167 assessed (Figure 6). Addition of matrigel significantly (*p* = 0.002) increased sensitivity of MCF‐7 cells (SF2 value of 0.25 ± 0.02 (MCF‐7 + M) vs. 0.35 ± 0.02 (MCF‐7)) to radiation alone, however, there was no significant difference in the radiosensitivity of MCF‐7 cells to AQ4N (*p* = 0.126) and KP167 (*p* = 0.260) treatment, +/−matrigel. MCF‐7 cells demonstrated similar radioresponse with SER_0.01_ values of 1.2 (AQ4N) and 1.5 (KP167) in both treatment conditions (+/−matrigel).

## 4 DISCUSSION

Hypoxia‐activated prodrugs (HAPs) are designed to be preferentially cytotoxic to hypoxic cancer cells which are largely associated with radio‐/chemo‐therapy failure.^35^ The newly developed HAP, KP167, comprises DNA intercalating and alkylating properties (Morral J., Aiyappa R., Sadiq M., Abdallah Q. M., Grau L., Puyol M. D., Shnyder S. D., Phillips R. M., Patterson L. H., Martin S. and Pors K., unpublished data). The latter provides a mode of action enabling KP167 to potentially be effective in targeting both quiescent (hypoxic) and proliferative cells, in contrast to AQ4N which is cytotoxic only in proliferating cells.^27^ This study assessed the single agent and radiosensitizing efficacy of KP167 in 2D and 3D models of luminal and TNBC cells.

Initially, the single agent efficacy of HAPs was assessed in luminal and TNBC cells grown in normoxia, hypoxia and spheroid cultures. Both AQ4N and KP167 were cytotoxic in all breast cancer spheroids and demonstrated a dose‐dependent reduction in cellular survival, suggesting that the presence of hypoxia in 3D spheroid cultures (≥500 μm) enabled activation of AQ4N and KP167 into their DNA binding cytotoxins. To confirm the hypoxia‐specific activation, breast cancer cells in 2D monolayers were made hypoxic at 1% O_2_, following which the single agent cytotoxicity of AQ4N and KP167 were assessed. For all experiments in 2D hypoxia (1% O_2_ for 48 h), carbonic anhydrase (CA9), the endogenous hypoxia marker,^36^, ^37^ was assessed for positive expression to ensure that cells being used in experiments were truly hypoxic. Compared with 3D cultures, increased cytotoxicity was observed with both AQ4N and KP167 reflecting the fact that cells grown as monolayers and uniformly subjected to hypoxia mediated efficient CYP‐mediated conversion of AQ4N and KP167 into their respective cytotoxic metabolites. This is in contrast to 3D spheroid cultures which contain normoxic, hypoxic and necrotic cells, all co‐existing in different layers of the spheroid structure and potentially demonstrating differential drug sensitivities.^38^ In normoxia, both AQ4N and KP167 treatment resulted in a dose dependent reduction in clonogenic survival. However, higher concentrations of HAPs were required to induce 50% cell killing in most cell lines. The triple negative MDAMB‐231 and MDAMB‐468 cells (>45 μM) were resistant to HAP treatment in normoxia and demonstrated the least reduction in survival. This finding is in agreement with previous studies which report minimal or no toxicity when AQ4N was tested against the NCI (National Cancer Institute) panel of 60 cell lines in normoxic conditions, where most cell lines had IC_50_ > 100 μM.^27^ In contrast, potent cytotoxicity was observed when AQ4N was bioreduced under hypoxic conditions, which suggests that efficient conversion of the drug into the DNA binding metabolite occurs in the absence of oxygen.^39^, ^40^ For example, Mehibel et al.,^41^ reported that the IC_50_ of AQ4N in macrophages was 40 μM in normoxia but gets reduced to 3.9 μM under hypoxic conditions, which suggests that AQ4N is bioreduced at a higher rate in hypoxic cells. Both AQ4N and KP167 treatments were cytotoxic in breast cancer cells grown in 2D hypoxia and 3D spheroid cultures when compared with response obtained in 2D normoxia, which may indicate the preferential hypoxia‐specific activation of these agents.^27^, ^42^

The cytochrome P450 proteins are a family of oxidative enzymes involved in the metabolism of many carcinogens and anti‐cancer agents.^43^ They are, due of their involvement in the metabolism of oestrogen and anti‐cancer agents used in breast cancer treatment, important in the development and progression of breast cancer [reviewed in^44^]. The cytochrome P450 enzymes CYP2S1 and CYP2W1 have been reported to be involved in the bioreductive activation of AQ4N into its cytotoxic metabolite, AQ4. AQ4N is reported to be more toxic in hypoxic cells, because of the competitive binding of oxygen to the active haem centre of CYP enzymes.^27^ We have shown that KP167 is anti‐proliferative in CYP2W1 transfected cells under hypoxia, suggesting involvement of CYP2W1 in the hypoxic bioactivation of KP167. Western blot data show that the highest expression of CYP2S1 and CYP2W1 was present in MDAMB‐436 and T47D cells, respectively, which may explain the increase in single agent cytotoxicity and greater radiosensitization in these cell lines, regardless of oxygen conditions. Such data demonstrate that higher expression of CYP2W1 may determine sensitivity to HAPs, even in the presence of oxygen. Apart from higher endogenous expression of CYP2S1, increased cytotoxicity observed in MDAMB‐436 cells may reflect the inherent BRCA‐1 deficient nature, which coupled with TOPO II inhibitors and/or DNA alkylators, might further decrease or completely inhibit its potential to repair DNA double‐strand breaks.^45^

Clinically, treatment of TNBCs continues to be challenging as there are, apart from PARP inhibitors, no routine clinically available targeted therapies to treat this breast cancer subtype. Adjuvant therapy in TNBCs rarely provides long‐term remission and the occurrence of metastasis is associated with high resistance to chemotherapy.^46^ Furthermore, regions of hypoxia and necrosis are higher in ER negative tumours, which is associated with local recurrence, radiotherapy resistance and higher rates of metastasis observed in this phenotype.^47^, ^48^ In the current study, the radiosensitivity obtained with KP167 in the triple negative MDAMB‐231 (SER_0.01_ 2.53), MDAMB‐468 (SER_0.01_ 2.28) and MDAMB‐436 (SER_0.01_ 4.55) spheroids were greater than that obtained with well‐established radiosensitisers such as KU‐55933 (SER_0.01_ 1.82‐MDAMB‐231, 1.68‐MDAMB‐468, 3.42‐MDAMB‐436) and Olaparib with SER_0.01_ of (1.62‐MDAMB‐231, 1.68‐MDAMB‐468, 2.35‐MDAMB‐436), with the latter trialled as a radiosensitiser in the RadioParp trial (NCT03109080).^49^ The increased cytotoxicity in TNBC cell lines may be because of shared characteristics with BRCA‐1 deficient tumours, commonly known as sporadic BRCAness.^50^ TNBCs may be susceptible to DNA repair inhibitors because these tumours have lower expression of BRCA‐1/2 or of genes involved in DNA repair pathways.^51^ Although further studies are required both in vitro and in vivo to confirm such findings, from the current study, KP167 appears to be a potent radiosensitiser in breast cancer and may hold promising potential, especially in the treatment of TNBCs. Liapis et al.,^52^ assessed the anti‐cancer efficacy TH‐302 in primary breast cancer and metastatic deposits in bone and reported that in combination with paclitaxel, TH‐302 was able to reduce tumour growth of primary breast cancers and metastatic deposits in bone (common site of breast cancer metastasis). Such data indicate that current HAPs can potentially be used both in a neoadjuvant and metastatic setting to target hypoxic breast cancers and improve patient outcomes. The greatest increase in radiosensitization was seen in 3D models of BRCA‐1 deficient MDAMB‐436 cells. Promising results from another pre‐clinical study demonstrated that in combination with radiation, the HAPs, PR‐104 and TH‐302 sensitize BRCA‐2 knockout mutants to radiation, indicating that these agents have the potential to exploit hypoxia as well as homologous recombination (HR) defects.^53^ This strategy holds true not only for BRCA‐1/2 deficient tumours, but hypoxic cancer cells demonstrate defective HR pathways, such as TNBC cells, which can be therapeutically exploited to induce greater radiosensitization.^54^, ^55^

In summary, the current study evaluated the role of DNA repair inhibitors and HAPs in modulating response of breast cancer cells to radiation. The novel HAP, KP167, was a highly effective hypoxia‐activated radiosensitiser across all three platforms (2D normoxia, hypoxia and 3D spheroids). In 3D spheroid cultures, radiosensitivity obtained in TNBCs was higher than previously observed with well‐established radiosensitisers such as KU‐55933, AQ4N and clinically approved agents such as Olaparib. Although further confirmation is required, the current study highlights KP167 as a potentially useful hypoxia‐activated radiosensitiser and suggests that it might be a promising agent, either single or combined with radiation, to improve outcome in breast cancers.

## AUTHOR CONTRIBUTIONS

**Radhika Aiyappa‐Maudsley:** Data curation (lead); formal analysis (lead); investigation (lead); methodology (lead); writing – original draft (lead); writing – review and editing (lead). **Lina Elsalem:** Investigation (supporting). **Ali I.M Ibrahim:** Resources (supporting). **Klaus Pors:** Resources (supporting); supervision (supporting); writing – review and editing (supporting). **Stewart G Martin:** Conceptualization (lead); funding acquisition (lead); investigation (lead); methodology (lead); project administration (lead); resources (lead); supervision (lead); writing – review and editing (lead).

## FUNDING INFORMATION

None to declare.

## CONFLICT OF INTEREST

The authors declare that they have no conflicts of interest.

## Supporting information


Appendix S1

## Data Availability

Data has not been previously shared.
