# Supplementary material for: In vitro radiosensitization of breast cancer with hypoxia‐activated prodrugs
Source: J Cell Mol Med. 2022 Jul 16;26(16):4577–90. doi: 10.1111/jcmm.17486 (PMC9357624; doi:10.1111/jcmm.17486)
Supplement: Supplementary file 1 — Appendix S1 [file JCMM-26-4577-s001.docx]

**Table S1 Cell culture media formulations**

| Cell line | Reagent | Source | Volume |
| --- | --- | --- | --- |
| MCF7  &  SW480 | RPMI-1640 medium  Heat Inactivated Iron Supplemented Donor Calf Serum  Penicillin/Streptomycin (with 10,000 units penicillin and 10 mg streptomycin/mL) | R8758, Sigma, UK  Gibco, Life Technologies, UK  P4333, Sigma, UK | 500 ml  50 ml  5 ml |
| T47D | Dulbecco’s Modified Eagle’s Medium - high glucose (with 4500 mg/L glucose, L-glutamine, and sodium bicarbonate, without sodium pyruvate)  Heat Inactivated Iron Supplemented Donor Calf Serum  Penicillin/Streptomycin (with 10,000 units penicillin and 10 mg streptomycin/mL) | D5796, Sigma, UK  Gibco, Life Technologies, UK  P4333, Sigma, UK | 500 ml  50 ml  5 ml |
| MDAMB-231  &  MDAMB-468 | Minimum essential medium eagle (MEM) (with Earle′s salts, nonessential amino acids and sodium bicarbonate, without L-glutamine)  Heat Inactivated Iron Supplemented Donor Calf Serum  Penicillin/Streptomycin (with 10,000 units penicillin and 10 mg streptomycin/mL)  L-Glutamine (200 mM)  MEM Non-essential amino acids solution (100x) | M2279, Sigma, UK  Gibco, Life Technologies, UK  P4333, Sigma, UK  G7513, Sigma, UK  M7145, Sigma, UK | 500 ml  50 ml  5 ml  5 ml  5 ml |
| MDAMB-436 | DMEM/F12 HEPES (with L-Glutamine and 15mM HEPES)  Heat Inactivated Iron Supplemented Donor Calf Serum  Penicillin/Streptomycin (with 10,000 units penicillin and 10 mg streptomycin/mL) | 31330, Invitrogen, UK  Gibco, Life Technologies, UK  P4333, Sigma, UK | 500 ml  5 ml  5 ml |

**Table S2 Radiobiologic parameters of breast cancer cells treated with IR and olaparib or KU-55933**

| Cell line | α  (Gy^-1^) | β  (Gy^-2^) | α/β  (Gy) | SF2 | SER _(0.01)_ |
| --- | --- | --- | --- | --- | --- |
| MCF-7  IR  Olaparib+IR  *t* test | 0.17±0.01  0.75±0.06  ***P*=0.0001** | 0.08±0.00  0.05±0.01  ***P*=0.021** | 2.20±0.37  16.38±5.66  ***P*=0.012** | 0.49±0.00  0.18±0.02  ***P*<0.00001** | 1.63±0.03 |
| T47D  IR  Olaparib+IR  *t* test | 0.24±0.12  0.70±0.06  ***P*=0.005** | 0.05±0.01  0.02±0.00  ***P*=0.043** | 5.12±3.82  26.10±2.27  ***P*=0.001** | 0.49±0.10  0.22±0.02  ***P*=0.011** | 1.61±0.18 |
| MDAMB-231  IR  Olaparib+IR  *t* test | 0.49±0.06  0.99±0.06  ***P*=0.0007** | 0.06±0.01  0.03±0.01  ***P*=0.045** | 7.77±2.67  31.75±16.50  *P*=0.067 | 0.31±0.04  0.10±0.02  ***P*=0.002** | 1.62±0.09 |
| MDAMB-468  IR  Olaparib+IR  *t* test | 0.36±0.05  1.00±0.09  ***P*=0.0005** | 0.04±0.00  0.01±0.00  ***P*=0.0001** | 7.52±1.70  164.67±79.19  ***P*=0.026** | 0.38±0.04  0.13±0.02  ***P*=0.0006** | 1.68±0.33 |
| MDAMB-436  IR  Olaparib+IR  *t* test | 0.54±0.06  1.56±0.06  ***P*=0.00002** | 0.04±0.01  0.04±0.06  *P*=0.493 | 15.62±1.00  38.25±6.91  ***P*=0.004** | 0.28±0.02  0.02±0.017  ***P*=0.0001** | 2.35±0.13 |

| Cell line | α  (Gy^-1^) | β  (Gy^-2^) | α/β  (Gy) | SF2 | SER _(0.01)_ |
| --- | --- | --- | --- | --- | --- |
| MCF-7  IR  KU-55933+IR  *t* test | 0.22±0.18  0.76±0.12  ***P*=0.013** | 0.08±0.02  0.03±0.01  ***P*=0.034** | 4.32±3.76  20.68±7.56  ***P*=0.022** | 0.41±0.13  0.19±0.04  ***P*=0.021** | 1.60±0.23 |
| T47D  IR  KU-55933+IR  *t* test | 0.17±0.11  0.63±0.09  ***P*=0.006** | 0.06±0.01  0.05±0.01  *P*=0.33 | 2.93±2.04  12.79±5.36  ***P*=0.04** | 0.55±0.10  0.21±0.04  ***P*=0.007** | 1.71±0.27 |
| MDAMB-231  IR  KU-55933+IR  *t* test | 0.37±0.24  1.08±0.04  ***P*=0.007** | 0.07±0.02  0.03±0.02  *P*=0.13 | 6.81±7.03  61.27±53.49  *P*=0.143 | 0.36±0.12  0.102±0.01  ***P*=0.020** | 1.82±0.21 |
| MDAMB-468  IR  KU-55933+IR  *t* test | 0.30±0.06  0.91±0.11  ***P*=0.005** | 0.05±0.01  0.01±0.00  ***P*=0.012** | 5.81±3.37  91.08±68.67  *P*=0.098 | 0.43±0.02  0.15±0.03  ***P*<0.001** | 1.68±0.33 |
| MDAMB-436  IR  KU-55933+IR  *t* test | 0.39±0.00  1.37±0.47  ***P*=0.028** | 0.04±0.01  0.01±0.00  ***P*=0.020** | 10.70±5.65  43.17±18.06  ***P*=0.041** | 0.35±0.08  0.03±0.02  ***P*=0.003** | 3.42±0.91 |

Statistical significance was determined by Student's *t*-test. The *P*-values are bold where they are ≤ 0.05. Abbreviations: IR, irradiation; SER_0.01_, sensitiser enhancement ratio at 1% survival; SF2, surviving fraction at 2Gy.

**
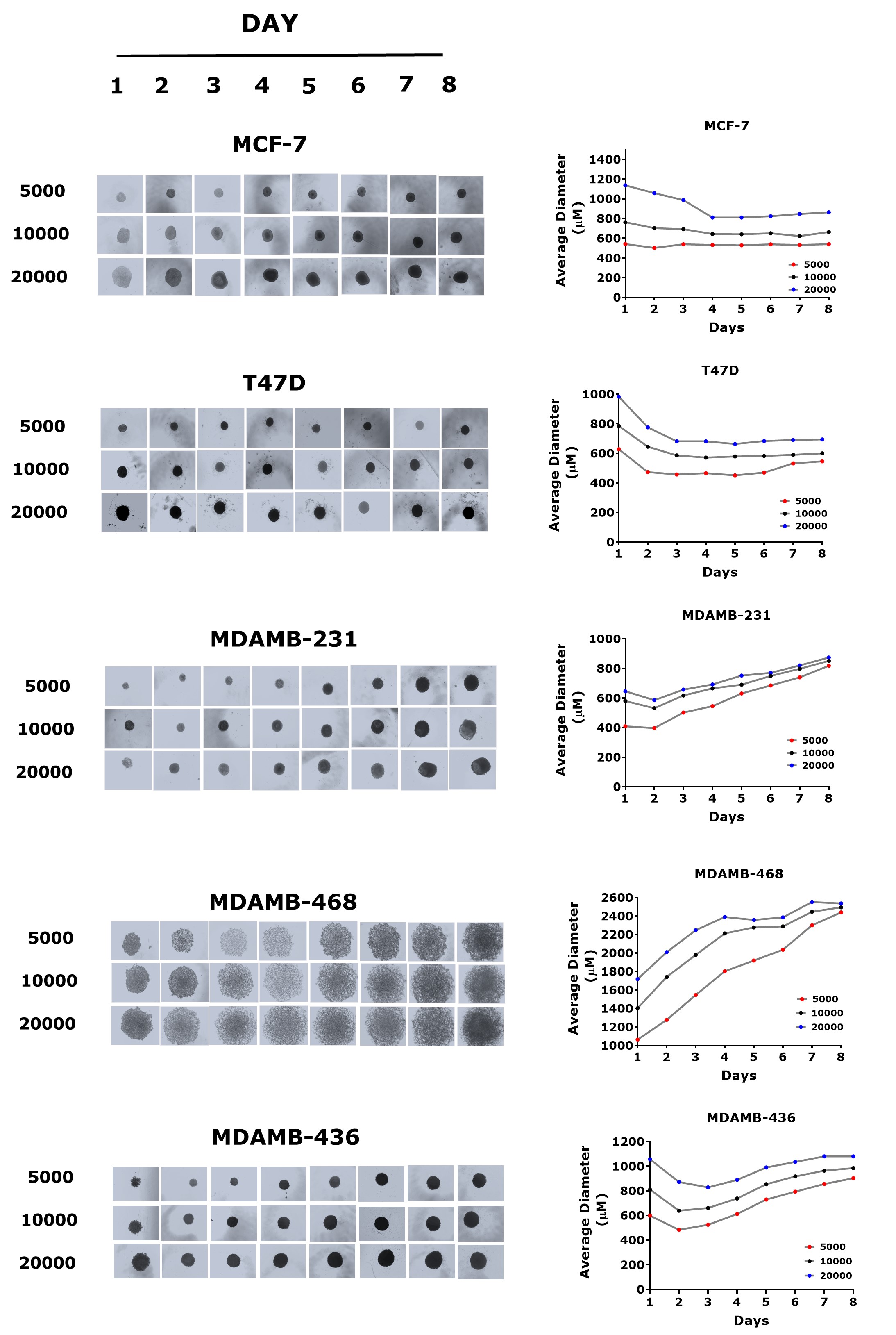
**

**Figure S1 Spheroid formation in breast cancer cells** A) Phase contrast micrographs of breast cancer spheroids from Day 1 to 8 with seeding density of 5000, 10,000 and 20,000 cells/well in 96-well ultra-low attachment plates. B) Spheroid diameter over an 8-day culture.

**
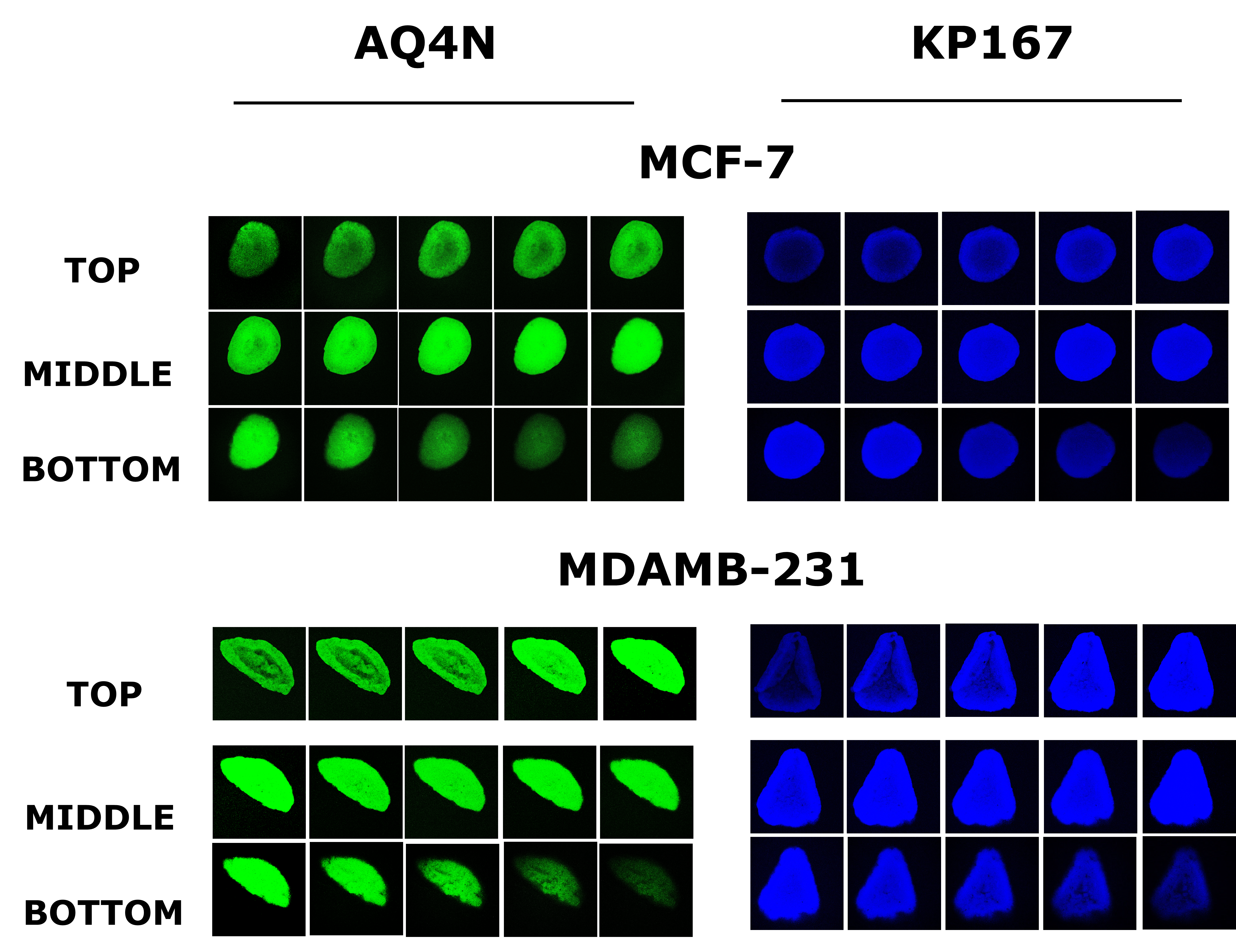
**

**Figure S2 Visualisation of AQ4N and KP167 penetration in MCF-7 and MDAMB-231 spheroids.** Representative images of spheroid cross-sections obtained using confocal microscope (10x magnification, step size 15 μm) are shown above. Experiments were repeated twice, with each containing 30 spheroids. Scale bar represents 200 μm.

**Table S3 Radiobiologic parameters of breast cancer cells treated with IR and AQ4N or KP167 in 2D normoxia**

| Cell line  Normoxia | α  (Gy^-1^) | β  (Gy^-2^) | α/β  (Gy) | SF2 | SER _(0.01)_ |
| --- | --- | --- | --- | --- | --- |
| MCF-7  IR  AQ4N+IR  *t* test | 0.14±0.05  0.32±0.10  ***P*=0.050** | 0.10±0.00  0.09±0.01  *P*=0.455 | 1.40±0.55  3.58±1.52  *P*=0.080 | 0.50±0.04  0.36±0.05  ***P*=0.024** | 1.17±0.06 |
| T47D  IR  AQ4N+IR  *t* test | 0.23±0.23  0.85±0.15  ***P*=0.019** | 0.07±0.02  0.02±0.01  *P*=0.043 | 4.40±5.35  32.72±19.54  *P*=0.072 | 0.44±0.10  0.16±0.04  ***P*=0.013** | 1.64±0.27 |
| MDAMB-231  IR  AQ4N+IR  *t* test | 0.42±0.19  0.66±0.02  *P*=0.174 | 0.06±0.02  0.06±0.02  *P*=0.904 | 7.50±4.73  12.15±6.87  *P*=0.389 | 0.33±0.10  0.20±0.04  *P*=0.125 | 1.29±0.28 |
| MDAMB-468  IR  AQ4N+IR  *t* test | 0.40±0.06  0.55±0.01  *P*=0.094 | 0.06±0.01  0.04±0.01  *P*=0.172 | 6.85±2.79  13.95±7.25  *P*=0.189 | 0.34±0.02  0.27±0.03  *P*=0.068 | 1.11±0.44 |
| MDAMB-436  IR  AQ4N+IR  *t* test | 0.50±0.24  0.65±0.24  *P*=0.366 | 0.05±0.00  0.04±0.03  *P*=0.932 | 10.26±3.05  24.81±28.02  *P*=0.422 | 0.30±0.03  0.23±0.07  *P*=0.252 | 1.19±0.12 |

| Cell line  Normoxia | α  (Gy^-1^) | β  (Gy^-2^) | α/β  (Gy) | SF2 | SER _(0.01)_ |
| --- | --- | --- | --- | --- | --- |
| MCF-7  IR  KP167+IR  *t* test | 0.14±0.05  0.29±0.13  *P*=0.148 | 0.10±0.01  0.10±0.02  *P*=0.778 | 1.40±0.55  3.04±2.16  *P*=0.270 | 0.50±0.04  0.36±0.06  ***P*=0.045** | 1.21±0.05 |
| T47D  IR  KP167+IR  *t* test | 0.23±0.23  1.07±0.12  ***P*=0.005** | 0.05±0.01  0.01±0.01  ***P*=0.005** | 5.12±3.82  175.47±79.57  ***P*=0.020** | 0.44±0.10  0.02±0.02  ***P*=0.006** | 1.93±0.09 |
| MDAMB-231  IR  KP167+IR  *t* test | 0.42±0.19  0.84±0.16  ***P*=0.050** | 0.06±0.02  0.06±0.03  *P*=0.991 | 7.50±4.73  21.75±23.00  *P*=0.352 | 0.33±0.10  0.14±0.02  ***P*=0.038** | 1.43±0.46 |
| MDAMB-468  IR  KP167+IR  *t* test | 0.40±0.06  0.67±0.21  *P*=0.108 | 0.06±0.01  0.04±0.02  *P*=0.499 | 6.85±2.79  19.54±17.57  *P*=0.284 | 0.34±0.02  0.21±0.06  ***P*=0.043** | 1.32±0.19 |
| MDAMB-436  IR  KP167+IR  *t* test | 0.64±0.00  1.09±0.02  ***P*<0.00001** | 0.01±0.00  0.03±0.03  *P*=0.412 | 32.94±7.02  71.23±73.24  *P*=0.271 | 0.23±0.01  0.08±0.01  ***P*=0.00002** | 1.68±0.08 |

Statistical significance was determined by Student's *t*-test. The *P*-values are bold where they are ≤ 0.05. Abbreviations: IR, irradiation; SER_0.01_, sensitiser enhancement ratio at 1% survival; SF2, surviving fraction at 2Gy.


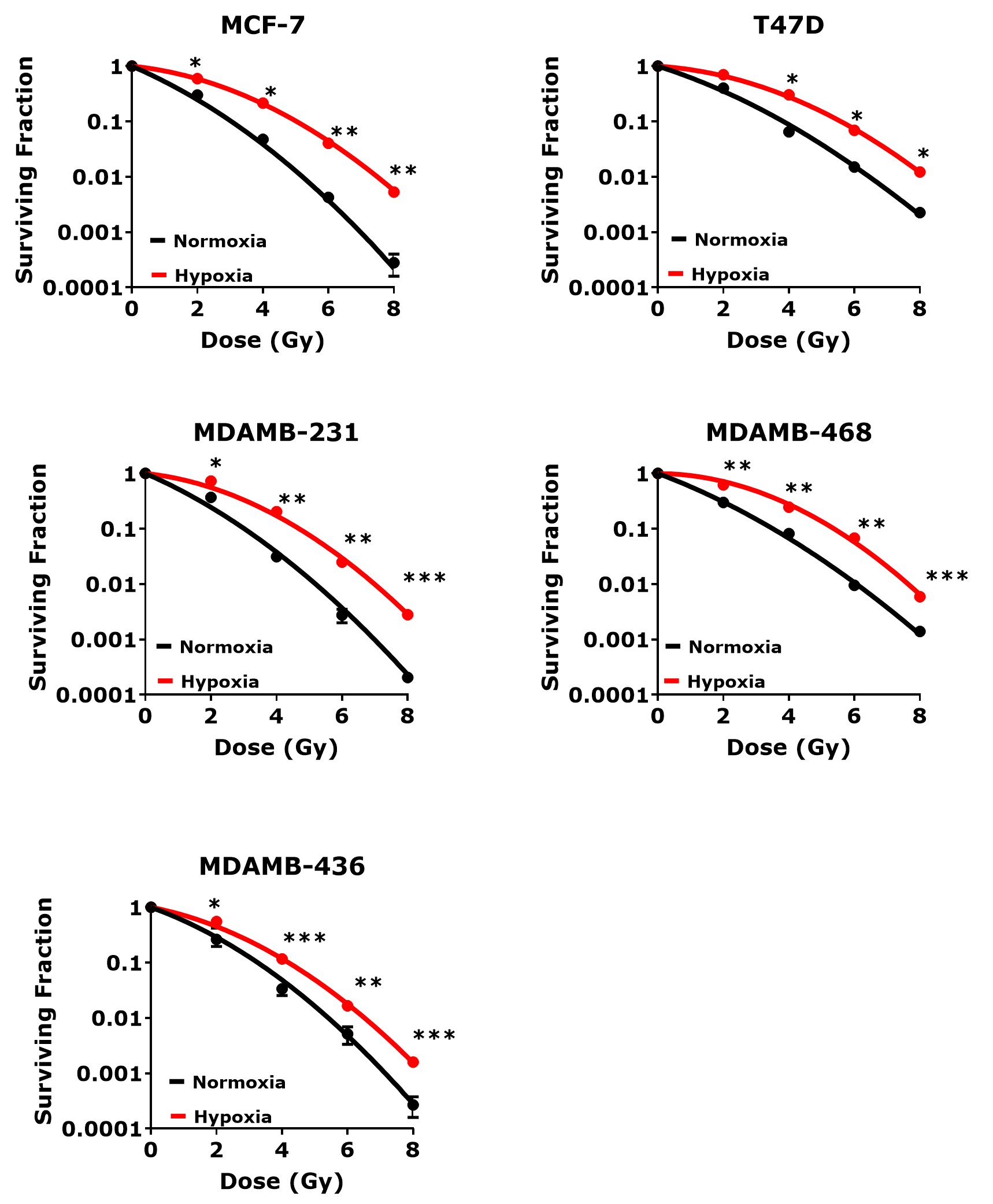


**Figure S3 Radiation response in 2D normoxia and hypoxia (1% O_2_).** PEs of individual cell lines under normoxia and hypoxia were: MCF-7 - 31 ± 1.1% and 23±1.0%; T47D - 18 ± 1.1% and 14±1.5%; MDAMB-231 - 56±4.0% and 44±2.6%; MDAMB-468 - 57 ± 6.1% and 42±5.8%; MDAMB-436 - 18±6.0% and 13±2.5%. Data represent the mean SF ± SD of three independent experiments, with each experiment containing six parallel data sets, analysed by Student’s *t*-test. **P*<0.05, ***P*<0.01, ****P*<0.001 for normoxia v hypoxia.

**Table S4 Radiobiologic parameters of breast cancer cells in 2D normoxia and hypoxia (1% O_2_) cultures.**

| Cell line  2D | α  (Gy^-1^) | β  (Gy^-2^) | α/β  (Gy) | SF2 | OER _(0.01)_ |
| --- | --- | --- | --- | --- | --- |
| MCF-7  2D Normoxia  2D Hypoxia  *t test* | 0.58±0.14  0.11±0.07  ***P=0.05*** | 0.05±0.01  0.08±0.03  *P=0.317* | 10.8±5.07  3.56±1.06  *P=0.187* | 0.24±0.05  0.58±0.03  ***P=0.019*** | 1.69±0.22 |
| T47D  2D Normoxia  2D Hypoxia  *t test* | 0.43±0.11  0.09±0.08  *P=0.07* | 0.04±0.02  0.05±0.01  *P=0.43* | 12.41±8.49  1.70±2.87  *P=0.22* | 0.35±0.49  0.66±0.09  ***P=0.05*** | 1.44±0.20 |
| MDAMB-231  2D Normoxia  2D Hypoxia  *t test* | 0.59±0.02  0.14±0.00  ***P=0.002*** | 0.05±0.00  0.07±0.01  ***P=0.022*** | 10.31±0.51  1.98±0.01  ***P=0.001*** | 0.24±0.01  0.55±0.01  ***P=0.002*** | 1.55±0.01 |
| MDAMB-468  2D Normoxia  2D Hypoxia  *t test* | 0.52±0.04  0.03±0.02  ***P=0.005*** | 0.04±0.01  0.08±0.01  ***P=0.010*** | 12.11±1.96  0.51±0.24  ***P=0.014*** | 0.30±0.01  0.71±0.02  ***P=0.002*** | 1.53±0.07 |
| MDAMB-436  2D Normoxia  2D Hypoxia  *t test* | 0.49±0.09  0.26±0.06  ***P=0.029*** | 0.06±0.01  0.06±0.00  *P=0.844* | 7.72±2.51  3.95±1.35  *P=0.084* | 0.28±0.04  0.44±0.05  ***P=0.013*** | 1.30±0.03 |

Statistical significance was determined by Student's *t*-test. The *P*-values are bold where they are ≤ 0.05. Abbreviations: IR, irradiation; OER_0.01_, oxygen enhancement ratio at 1% survival; SF2, surviving fraction at 2Gy.

**Table S5 Radiobiologic parameters of breast cancer cells in 2D hypoxia treated with IR and AQ4N or KP167 in 2D hypoxia**

| Cell line  2D Hypoxia | α  (Gy^-1^) | β  (Gy^-2^) | α/β  (Gy) | SF2 | SER _(0.01)_ |
| --- | --- | --- | --- | --- | --- |
| MCF-7  IR  AQ4N+IR  *t* test | 0.35±0.09  0.62±0.10  ***P*=0.028** | 0.04±0.01  0.02±0.01  *P*=0.092 | 8.24±3.29  27.86±18.73  *P*=0.148 | 0.41±0.05  0.25±0.04  ***P*=0.020** | 1.30±0.08 |
| T47D  IR  AQ4N+IR  *t* test | 0.06±0.05  0.33±0.34  *P*=0.088 | 0.06±0.01  0.11±0.06  ***P*=0.0001** | 1.01±0.94  6.71±9.74  ***P*=0.0006** | 0.68±0.05  0.35±0.17  ***P*<0.0001** | 1.74±0.32 |
| MDAMB-231  IR  AQ4N+IR  *t* test | 0.27±0.13  0.72±0.10  ***P*=0.010** | 0.06±0.01  0.07±0.03  *P*=0.764 | 4.77±3.61  12.19±6.93  *P*=0.175 | 0.45±0.09  0.18±0.03  ***P*=0.008** | 1.69±0.11 |
| MDAMB-468  IR  AQ4N+IR  *t* test | 0.19±0.04  0.29±0.16  *P*=0.360 | 0.06±0.01  0.06±0.02  *P*=0.873 | 2.98±1.01  5.93±5.77  *P*=0.433 | 0.52±0.03  0.43±0.10  *P*=0.225 | 1.13±0.10 |
| MDAMB-436  IR  AQ4N+IR  *t* test | 0.63±0.03  1.29±0.16  ***P*=0.002** | 0.24±0.40  0.01±0.01  *P*=0.390 | 52.15±23.91  270.76±365.19  *P*=0.359 | 0.26±0.01  0.07±0.02  ***P*=0.0003** | 1.94±0.25 |

| Cell line  2D Hypoxia | α  (Gy^-1^) | β  (Gy^-2^) | α/β  (Gy) | SF2 | SER _(0.01)_ |
| --- | --- | --- | --- | --- | --- |
| MCF-7  IR  KP167+IR  *t* test | 0.30±0.09  0.73±0.06  ***P*=0.034** | 0.05±0.01  0.04±0.01  *P*=0.445 | 5.52±2.75  19.44±10.13  *P*=0.201 | 0.43±0.06  0.19±0.13  ***P*=0.032** | 1.56±0.12 |
| T47D  IR  KP167+IR  *t* test | 0.04±0.01  0.94±0.09  ***P*=0.006** | 0.06±0.01  0.01±0.01  ***P*=0.021** | 0.63±0.29  384.44±476.85  *P*=0.372 | 0.69±0.02  0.14±0.02  ***P*=0.001** | 2.37±0.11 |
| MDAMB-231  IR  KP167+IR  *t* test | 0.30±0.01  0.90±0.06  ***P*=0.005** | 0.05±0.00  0.02±0.00  ***P*=0.009** | 53.98±34.91  44.44±13.48  ***P*=0.055** | 0.43±0.01  0.15±0.01  ***P*=0.003** | 1.75±0.04 |
| MDAMB-468  IR  KP167+IR  *t* test | 0.22±0.01  0.81±0.00  ***P*=0.0001** | 0.05±0.00  0.01±0.00  ***P*=0.002** | 3.83±0.19  106.01±42.31  *P*=0.076 | 0.50±0.00  0.18±0.00  ***P*=0.0002** | 1.72±0.02 |
| MDAMB-436  IR  KP167+IR  *t* test | 0.52±0.08  0.93±0.22  ***P*=0.04** | 0.01±0.01  0.05±0.03  *P*=0.168 | 39.36±26.23  29.05±29.24  *P*=0.672 | 0.32±0.03  0.12±0.03  ***P*=0.003** | 1.81±0.33 |

Statistical significance was determined by Student's *t*-test. The *P*-values are bold where they are ≤ 0.05. Abbreviations: IR, irradiation; SER_0.01_, sensitiser enhancement ratio at 1% survival; SF2, surviving fraction at 2Gy

**Table S6 Radiobiologic parameters of breast cancer spheroids treated with IR and/or AQ4N or KP167in spheroids.**

| Cell line  Spheroids | α  (Gy^-1^) | β  (Gy^-2^) | α/β  (Gy) | SF2 | SER _(0.01)_ |
| --- | --- | --- | --- | --- | --- |
| MCF-7  IR  AQ4N+IR  *t* test | 0.46±0.08  0.57±0.15  *P*=0.326 | 0.03±0.01  0.03±0.01  *P*=0.527 | 12.89±5.46  25.69±22.32  *P*=0.389 | 0.36±0.02  0.22±0.02  ***P*=0.002** | 1.30±0.08 |
| T47D  IR  AQ4N+IR  *t* test | 0.60±0.18  1.12±0.21  ***P*=0.035** | 0.01±0.02  0.01±0.02  *P*=0.396 | 26.09±19.73  280.15±36.3  *P*=0.295 | 0.26±0.07  0.10±0.03  ***P*=0.027** | 1.58±0.06 |
| MDAMB-231  IR  AQ4N+IR  *t* test | 0.6±0.06  0.99±0.04  ***P*=0.008** | 0.15±0.19  0.01±0.01  *P*=0.276 | 8.83±7.98  95.89±50.00  ***P*=0.040** | 0.25±0.02  0.13±0.01  ***P*=0.007** | 1.44±0.04 |
| MDAMB-468  IR  AQ4N+IR  *t* test | 0.46±0.06  0.95±0.10  ***P*=0.002** | 0.04±0.01  19.03±4.89  *P*=0.77 | 9.95±3.29  19.03±4.89  *P*=0.056 | 0.32±0.03  0.27±0.03  ***P*=0.0008** | 1.64±0.08 |
| MDAMB-436  IR  AQ4N+IR  *t* test | 0.03±0.03  1.73±0.14  ***P*=0.0005** | 0.09±0.01  0.05±0.01  *P*=0.415 | 0.35±0.38  32.75±2.15  ***P*=0.004** | 0.62±0.02  0.12±0.01  ***P*=0.006** | 3.33±0.18 |

| Cell line  Spheroids | α  (Gy^-1^) | β  (Gy^-2^) | α/β  (Gy) | SF2 | SER _(0.01)_ |
| --- | --- | --- | --- | --- | --- |
| MCF-7  IR  KP167+IR  *t* test | 0.43±0.04  0.83±0.11  ***P*=0.004** | 0.04±0.01  0.01±0.01  ***P*=0.026** | 10.51±2.31  102.04±70.49  *P*=0.087 | 0.35±0.02  0.18±0.03  ***P*=0.002** | 1.46±0.66 |
| T47D  IR  KP167+IR  *t* test | 0.69±0.15  1.38±0.12  ***P*=0.004** | 0.02±0.01  0.01±0.01  *P*=0.626 | 20.83±26.73  165.50±184.86  *P*=0.250 | 0.23±0.05  0.06±0.01  ***P*=0.009** | 1.76±0.20 |
| MDAMB-231  IR  KP167+IR  *t* test | 0.70±0.12  2.04±0.25  ***P*=0.001** | 0.02±0.01  0.06±0.03  *P*=0.202 | 14.81±4.38  35.91±13.03  *P*=0.056 | 0.21±0.04  0.02±0.01  ***P*=0.001** | 2.53±0.06 |
| MDAMB-468  IR  KP167+IR  *t* test | 0.46±0.06  1.57±0.06  ***P*=0.00003** | 0.04±0.008  0.01±0.01  ***P*=0.016** | 9.95±3.29  87.16±75.79  *P*=0.152 | 0.27±0.03  0.04±0.01  ***P*=0.0002** | 2.28±0.24 |
| MDAMB-436  IR  KP167+IR  *t* test | 0.03±0.01  2.22±0.19  ***P*=0.004** | 0.09±0.00  0.10±0.02  *P*=0.595 | 0.34±0.06  -21.58±2.86  ***P*=0.008** | 0.64±0.00  0.01±0.01  ***P*=0.0001** | 4.55±0.24 |

Statistical significance was determined by Student's *t*-test. The *P*-values are bold where they are ≤ 0.05. Abbreviations: IR, irradiation; SER_0.01_, sensitiser enhancement ratio at 1% survival; SF2, surviving fraction at 2Gy.

**Table S7 Compiled SER_0.01_ values of DNA repair inhibitors and hypoxia-activated prodrugs.**

| Cell lines | SER_0.01_ | | | | | | | | |
| --- | --- | --- | --- | --- | --- | --- | --- | --- | --- |
|  | **Olaparib** | **KU-55933** | **AQ4N** | | | **KP167** | | | |
|  | 2D Normoxia | 2D Normoxia | 2D Normoxia | 2D  Hypoxia | 3D Spheroids | 2D Normoxia | 2D  Hypoxia | 3D Spheroids |  |
| MCF-7 | 1.63±0.03 | 1.60±0.23 | 1.17±0.06 | 1.30±0.08 | 1.30±0.08 | 1.21±0.05 | 1.56±0.12 | 1.46±0.66 |  |
| T47D | 1.61±0.18 | 1.71±0.27 | 1.64±0.27 | 1.74±0.32 | 1.58±0.06 | 1.93±0.09 | 2.37±0.11 | 1.76±0.20 |  |
| MDAMB-231 | 1.62±0.09 | 1.82±0.21 | 1.29±0.28 | 1.69±0.11 | 1.44±0.04 | 1.43±0.46 | 1.75±0.04 | 2.53±0.06 |  |
| MDAMB-468 | 1.68±0.33 | 1.68±0.33 | 1.11±0.44 | 1.13±0.10 | 1.64±0.08 | 1.32±0.19 | 1.72±0.02 | 2.28±0.24 |  |
| MDAMB-436 | 2.35±0.13 | 3.42±0.91 | 1.19±0.12 | 1.94±0.25 | 3.33±0.18 | 1.68±0.08 | 1.81±0.33 | 4.55±0.24 |  |

Abbreviations: SER_0.01_, sensitiser enhancement ratio at 1% survival.
